# A cranial approach to transplastron coeliotomy and gastrotomy performed in a Sri Lanka Black Turtle (*Melanochelys trijuga thermalis*): Case Report

**DOI:** 10.1002/ccr3.6436

**Published:** 2022-11-05

**Authors:** Bope Gamage Sashikala Sewwandi Gamage, Bulathge Vijitha Pushpakumara Perera, Kashmini Kaushalya Sumanasekera, Kalani Piyumika Samarakoon, Vithana Pathirannehalage Malaka Kasun Abeywardhane, Ayona Silva‐Fletcher

**Affiliations:** ^1^ Department of Wildlife Conservation Elephant Transit Home Udawalawe Sri Lanka; ^2^ Department of National Zoological Gardens Elephant Orphanage Pinnawala Sri Lanka; ^3^ Department of Wildlife Conservation Elephant Holding Centre Udawalawe Sri Lanka; ^4^ The Royal Veterinary College University of London London UK

**Keywords:** foreign body, general surgery, *Melanochelys trijuga thermalis* (black turtle), osteotomy, transplastron coeliotomy, veterinary

## Abstract

Successful transplastron coeliotomy via a temporary cranial plastron osteotomy can be conducted in turtles to remove foreign bodies lodged in the stomach, using basic equipment. A year later, the turtle was returned to the wild indicating that major surgeries with complete recovery can be achieved in this species.

## Graphical abstract

A foreign body lodged in the stomach of a turtle can be removed surgically through cranial approach of the trasplastroncoeliotomy, using basic surgical equipment.

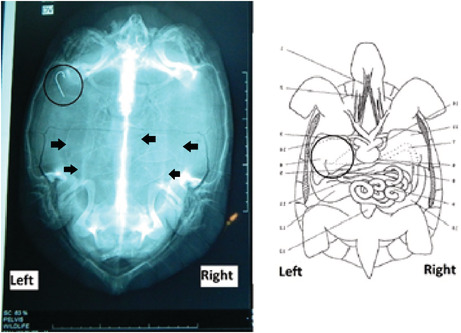

## 1 INTRODUCTION

The subspecies, *Melanochelys trijuga thermalis* (Sri Lanka black turtle) belongs to the order Testudines, family Geoemydidae and genus Meanochelys. The species *Melanochelys trijuga* is classified as a near threatened species in the IUCN Red list.^1^ It is a species of medium size freshwater turtle usually recorded in India, Maldives, and Sri Lanka. The Parker's black turtle (*Melanochelys trijuga pakeri*) black turtle (*Melanochelys trijuga thermalis*), and soft or flapshell turtle (*Lissemys punctata punctata*) are three freshwater turtle species found in Sri Lanka. The Star tortoise (*Geochelone elegans*) is the only terrestrial turtle identified in Sri Lanka.^2^

Clinical and surgical conditions associated with terrestrial turtle species are different from freshwater species. Upper respiratory diseases commonly occur among both fresh water and terrestrial species, but the etiological factors are not the same.^3^ Shell rot, oral herpes virus infection, skin sloughing, or peeling conditions associated with hypervitaminosis, internal infections, abscesses, impactions, foreign body ingestions, reproductive problems, ocular diseases, and internal and external parasitism are the other health‐related problems found in turtles.^4^, ^5^

Chelonians are popular as pet animals, therefore, most of the reported diseases and surgical cases are related to captive turtles. Disorders involving shell, such as deformities associated with nutritional factors (excess dietary proteins, excess, or deficient minerals and vitamins, metabolic bone diseases) and traumatic injuries can be found ordinarily in turtles.^5^ Anthropogenic causes such as traumatic injuries and foreign body obstructions are the common health‐related instances in turtles, reported in Sri Lanka (personal communication; B. V. P. Perera) because these animals are not common in captivity. Fresh water and marine turtles are more prone to foreign body ingestion of fishhooks, gravel, and plastic objects that float on the water. Surgery is the only option in most of the cases diagnosed with sharp or pointed object ingestion^4^ when sophisticated facilities, such as endoscopy, are unavailable. Other than this, there are few other common indications for coeliotomy in a turtle. These include removal of cystic calculi, correction of egg binding, repair of traumatic injuries, exploratory coeliotomy, and peritonitis cleanup.^6^, ^7^, ^8^

## 2 CASE HISTORY AND EXAMINATION

A free‐living black turtle with a nylon thread extending from the oral cavity was found by a fisherman in the Udawalawe region, Sri Lanka, on 26 January 2020. The turtle was brought to the wild animal hospital at the Elephant Transit Home (ETH) for investigation and treatment in the evening of the same day. It was an adult female with a body weight of 2.77 kg and other measurements as recorded in Table 1.

**TABLE 1:** The body measurements of the black turtle that was brought to the wild animal hospital at the Elephant Transit Home, Udawalawe, Sri Lanka

01	Carapace length (curved)	30.5 cm
02	Carapace length (straight)	29 cm
03	Carapace width (curved)	26.5 cm
04	Carapace width (straight)	19 cm
05	Plastron length	27 cm
06	Plastron width	15.7 cm
07	Shell height	11 cm
08	Body weight	2.77 kg
09	The number of growth rings on a scute	11

**FIGURE 1:**
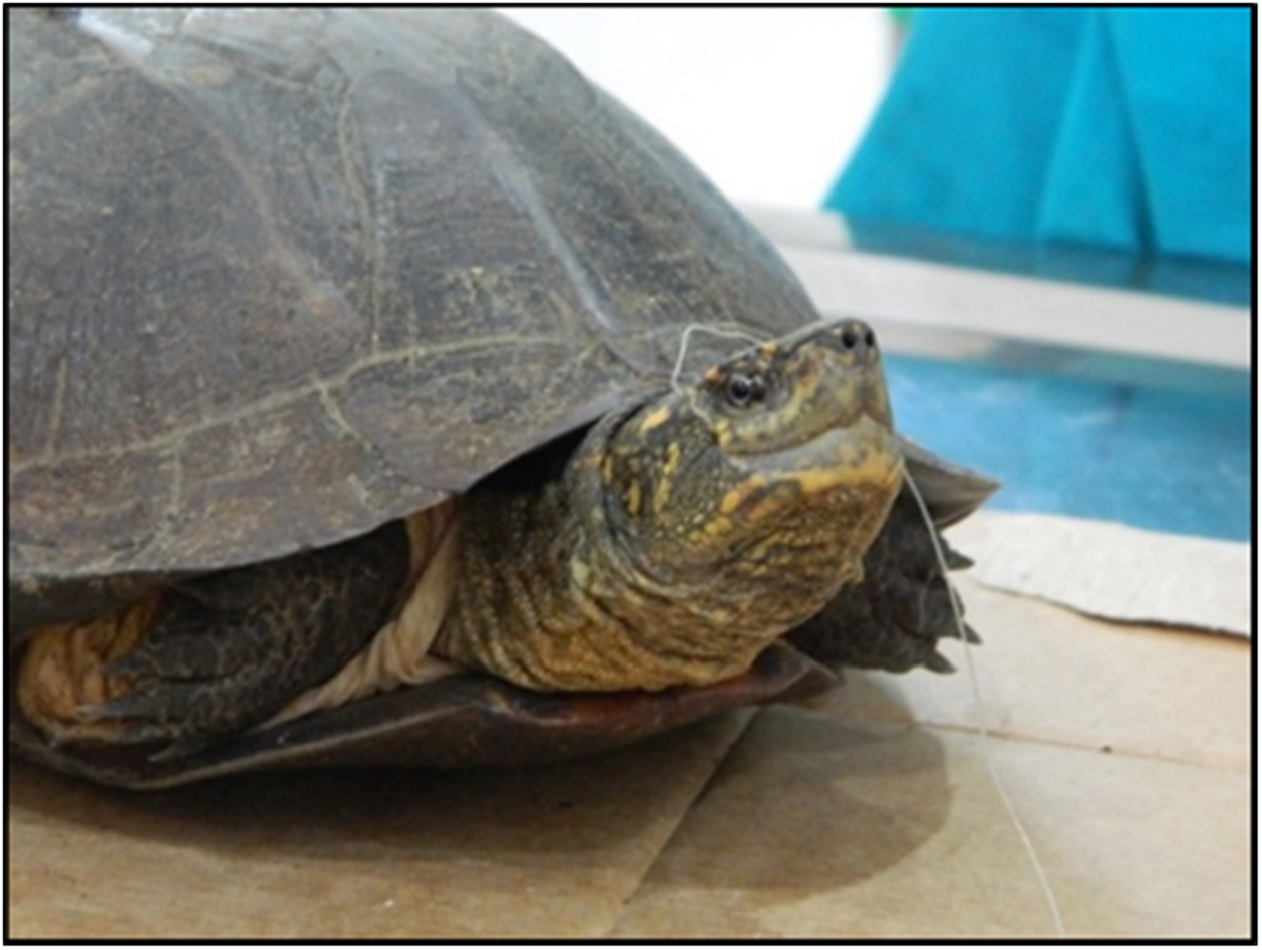
Black Turtle with the thread, at the presentation

An attempt to age the turtle estimated that it could be 11 years old, based on the number of growth plates. However, according to Bertolero and others in 2005, the number of growth plates is not precise in age determination. The reptile was examined thoroughly to identify an origin or any attachment to the thread, such as a hook (Figure 1), but any hook or hook attached site could not be detected by external physical examination. Moreover, the thread could not be easily dislodged by traction. The patient was observed overnight, and food and water were provided to assess its' appetite and any dysphagia while ingestion. The turtle did not show any signs or interest in food or water on the day of presentation and overnight.

## 3 DIFFERENTIAL DIAGNOSIS, INVESTIGATIONS, AND TREATMENT

On the following morning, radiographs of the dorso‐ventral and ventro‐dorsal views of the patient were taken to observe the presence and position of a possible hook attached to the thread.

**FIGURE 2:**
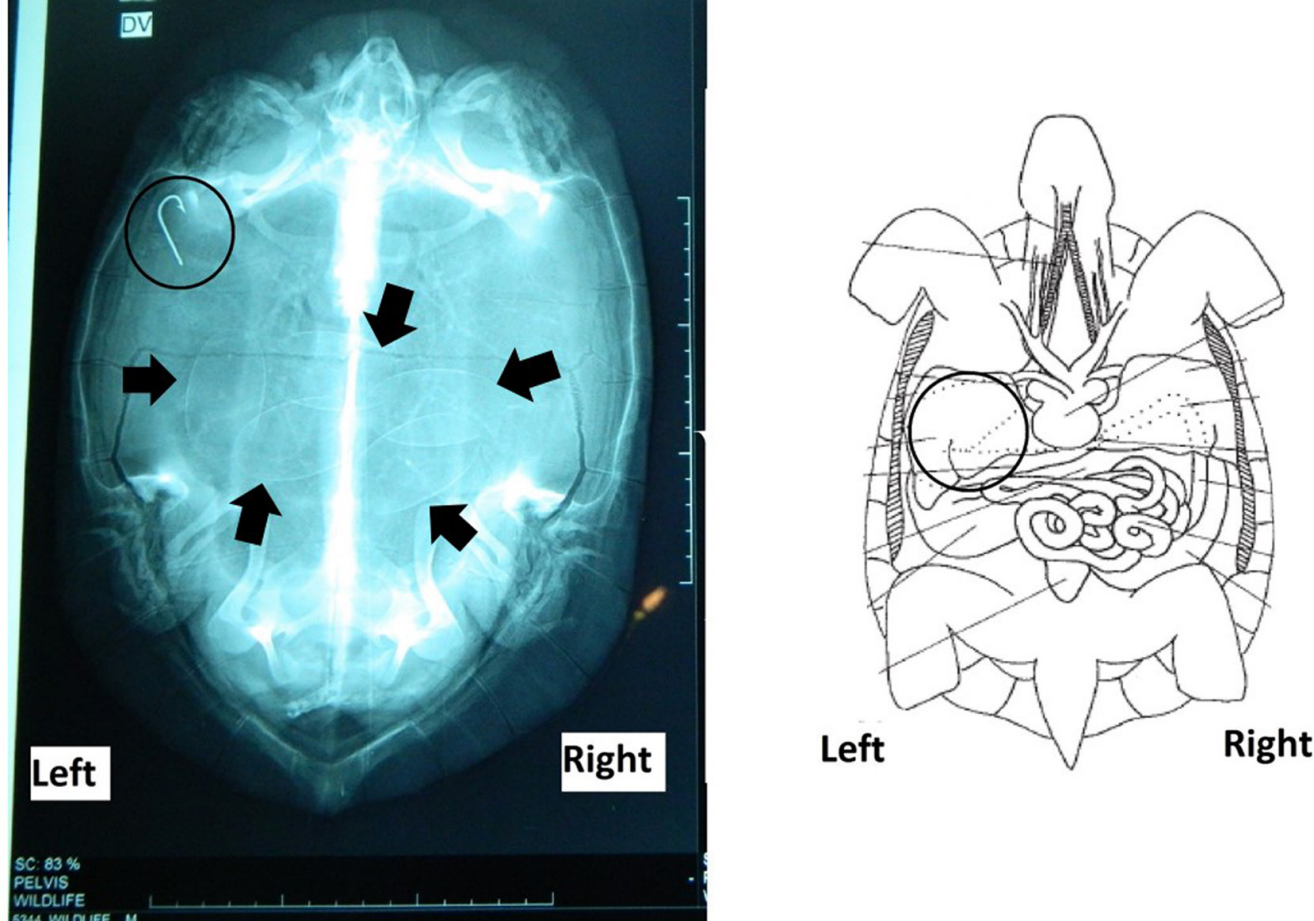
X‐ray and schematic diagram shows the location of the hook in the stomach (see encircled) and the presence of five eggs (see arrows)

Radiographs revealed a hook on the cranial left corner of the body, which is the exact position of the stomach (Figure 2). Considering the anatomy of the terrapin, it was decided to perform a coeliotomy and a gastrotomy to remove the hook before the deterioration of its condition. Other than the hook, the presence of five oval‐shaped eggs were observed in the radiograph.

Following the diagnosis of the position of the hook, a surgical approach was taken as the primary treatment. To prepare the surgical site, the patient was first anesthetized. Anesthesia was induced with intramuscular (pectoral muscles) 25 mg/kg ketamine hydrochloride (Ketamin; Dutch Farm International; 100 mg/ml) and 0.5 mg/kg diazepam (Centurion Healthcare; 5 mg/ml). Anesthesia was maintained with ketamine hydrochloride intramuscular (pectoral muscles under the fore limbs) injections, given twice, approximately at 60‐min intervals. Unfortunately, intravenous access could not be maintained throughout the surgery. Ketoprofen (Korea thumb vet; 100 mg/ml) 0.2 mg/kg intramuscular was given as an analgesic and enrofloxacin (Enrodac 10 vet; Zydus cadila vet; 100 mg/ml; 10 mg/kg) intramuscular as a prophylactic antibiotic prior to the surgery. The vital parameters were monitored during the surgery and the patient was intubated (uncuffed size 4.5 mm I.D. endotracheal tube) to assist in an emergency if artificial ventilation was required, however, it was not performed as the average respiratory rate was 0.66 per minute throughout the surgery.

**FIGURE 3:**
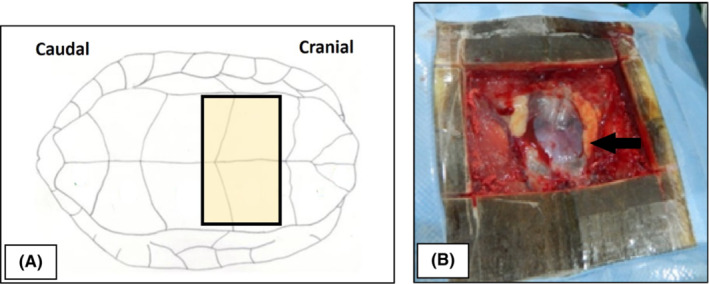
(A) Schematic diagram shows the surgical site (brown square) of approach and (B) an image of the surgical site, which is close to the heart (black arrow)

The site of approach for the surgery is shown in the schematic diagram (Figure 3A). A more cranial surgical approach from the plastron was decided due to the position of the hook, as revealed by the radiograph. This procedure had to be undertaken with caution, as the heart is located cranial and next to the stomach. The turtle was positioned in dorsal recumbency for the transplastron coeliotomy approach. A temporary osteotomy was performed over the ventrum to reach the stomach. The outline of the surgical site was made on the plastron using the point of a scalpel blade. The surgical site was properly cleaned with soap and water and scrubbed using 10% isopropyl alcohol and 1% povidone iodine.

The plastron was incised, following the lateral, cranial, and caudal plastron incision outlines. A hand saw blade held at a 45‐degree angle to the plastron surface was used to make the incision and extreme care was taken to minimize any soft tissue damage. With the cut plastron in place, soft tissues that were attached to the underside of the plastron were detached carefully by blunt dissection. Then the plastron flap was completely excised (Figure 3B) and was covered by a moistened sterile gauze.

**FIGURE 4:**
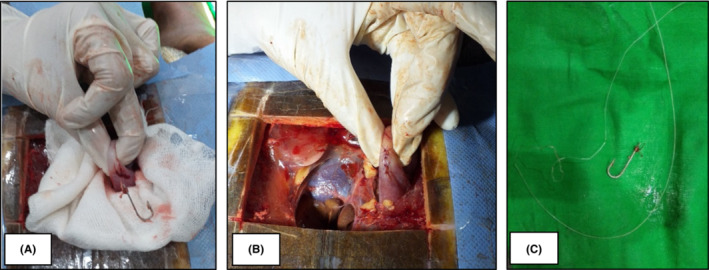
(A) Ingested fish hook inside the stomach, (B) Sutured stomach wall after removal of hook, and (C) Removed fish hook and thread

Through blunt dissection with hemostat forceps, the stomach was approached on left side, without damaging the major vessels such as the left pulmonary vein, artery, and descending aorta lateral to the heart. The hook was located by palpating the stomach, and a 3–5 mm long surgical incision was made over the greater curvature of the stomach, identifying an avascular area. The area surrounding the incision was covered with sterile gauze to prevent contamination of coelomic structures, from the gastric content. The hook was removed from the incision (Figure 4A) and the thread was cut at the point of attachment to the hook. The remaining part of the thread was removed by traction through the mouth. The incision was closed stepwise using a two layer‐approach. First, the sub mucosal and muscular layers of the stomach were closed (Figure 4B) using a simple interrupted suture pattern with 3–0 polygalactin 910 suture material. Then a simple continuous suture pattern was used to close the serosal surface of the gastric wall, with the same suture material. The hook and the thread which were removed are shown in Figure 4C.

**FIGURE 5:**
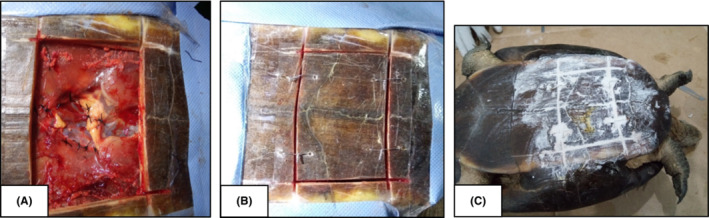
(A) Sutured bluntly dissected sites after the surgery, (B) stabilizing sutures placed through predrilled holes with orthopedic wires to anchor the plastron flap to its original position, (C) Plaster of Paris used to cover the gap between cut edges

**FIGURE 6:**
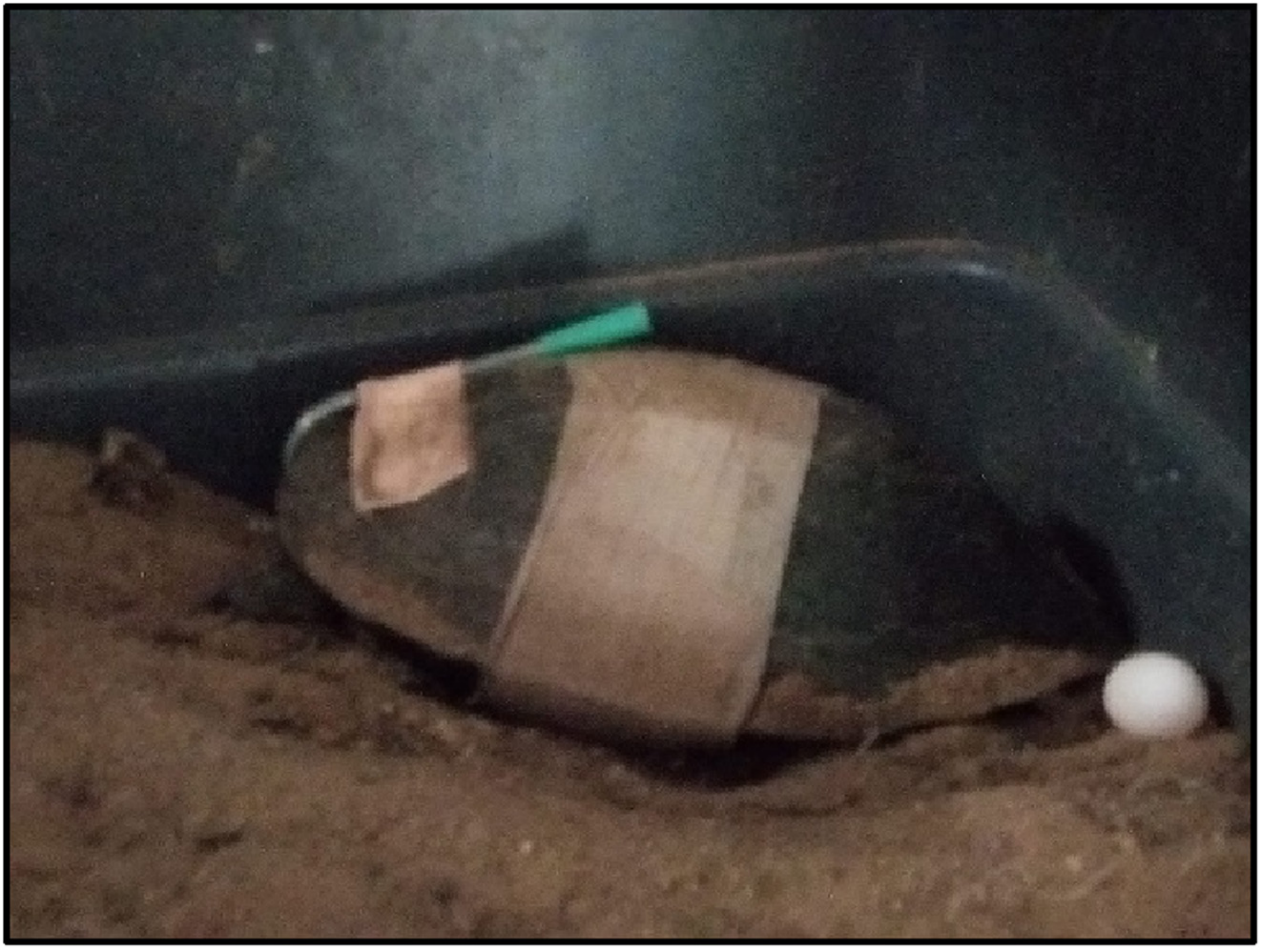
Tortoise with the oesophagostomy tube; the dressing placed to cover the surgical site; laid eggs

Prior to the repositioning of the stomach, the site of the incision and the stomach surface were washed with normal saline (0.9%). Bluntly dissected sites were sutured with simple interrupted sutures, using a 3–0 polygalactin 910 (Figure 5A). Four stabilizing sutures were placed through predrilled holes, with orthopedic wires, to anchor the plastron flap to its original position (Figure 5B). Plaster of Paris was used on top of the plastron incision temporarily to prevent contamination, as a more suitable material like epoxy was not available (Figure 5C). Duration of the whole surgical procedure was approximately 4 h. A dressing with an absorbent pad was placed over the surgical site (Figure 6).

## 4 OUTCOME AND FOLLOW‐UP

Antibiotic and analgesic medicines were administered for a week. Food was withheld for 48 h, while normal saline (0.9%) was administered subcutaneously and intracoelomically. Mild serosanguinous discharge was evident from the incision, during the first 3 days following the surgery, later it reduced and ceased. Hence, no evidence of infection was observed.

The turtle remained inappetent after the first 2 days post‐surgery, given the immature eggs in her abdomen it was felt a stomach tube would be beneficial for feeding. An oesophagostomy was performed (Figure 6) and the turtle was given 15–20 ml of liquidized fish per day through the oesophagostomy tube. The volume was decided upon the basic rule of feeding chelonians, which is 1%–2% of body weight every 24 h. Although a small amount was fed initially, it was gradually increased later.^9^ Tube feeding was continued for about a month. During the post‐operative period, the turtle showed signs of recovery, started drinking water, and freely moved within the cage. The most successful outcome of the surgery was observed 1 month later, when the turtle started to lay eggs. Within 8 weeks following the surgery, the animal laid all five eggs that were observed in the radiographs, and it covered the eggs with sand using its back legs (Figure 6). However, none of the eggs hatched.

Ninety days later, the temporary plaster of Paris cover was removed and replaced with epoxy putty and introduced to a water pond for swimming. A year later, it was released back to a natural pond in the Udawalawe national park, near ETH. Unfortunately, the turtle could not be tagged before releasing back to the wild, but a turtle that is suspected to be the particular turtle could be observed regularly near the released point.

## 5 DISCUSSION

Wildlife can sustain injuries due to human activities and some animals may require unusual approaches to resolve the problems and to be treated back to full recovery. To the best of our knowledge, the surgery performed on this turtle was a first in Sri Lanka, thus, there were many issues to consider. Turtles have a slow rate of respiration, thus it can become slower or apnoeic during anesthesia, because they cannot move their limbs to alter the volume of coelomic cavity necessary for respiration.^3^ Therefore, endotracheal intubation is vital to provide mechanical ventilation if required. The glottis of chelonians can be easily visualized, as it is located at the base and top of the fleshy tongue. Moreover, the availability of oxygen and positive pressure ventilation are essential factors to consider before the surgery.

In transplastron surgery, the patient should be positioned in dorsal recumbency, which can also directly affect respiration. In this position, all the abdominal organs will lay on the lung tissue, exerting extra pressure.^10^ Therefore, sandbags or foam supports can be used to elevate the head and neck, to ensure the passage of air to the lungs and support the limbs to prevent pressure necrosis.^6^ In literature, three sides of the plastron flap were completely cut, and the other side was partially incised to elevate the flap, but this may hinder access to the surgical site during the procedures on the internal organs, such as stomach.^11^ Therefore, during this surgery, the plastron flap was completely removed to get access to the stomach.

Hemorrhages are observed as a common complication that can occur during surgery. Blood loss between 0.4–0.8 ml blood per 100 g of body weight can be tolerated by a healthy tortoise.^6^

The specialty of the procedure, in this case, is the more cranial position of the surgical site, which was performed to aid access to the stomach. In previously published articles, transplastron coeliotomy incisions have been made on a mid or more caudal position.^6^, ^10^ However, in this novel procedure, a careful approach is necessary to prevent pericardial trauma. Incisions made on the caudal aspects of the plastron are usually used for the removal of urinary calculi and surgeries of the reproductive tract. In such cases, only two main vessels (ventral abdominal veins) are encountered,^4^ but when the approach is more cranial and towards the left, it is necessary to consider three main vessels (left pulmonary artery, left systemic arch, and left pulmonary vein).^10^, ^12^ Therefore, extra care was taken during the procedure to prevent damages to the blood vessels.

An oscillating sagittal saw is the ideal instrument to cut open the plastron. In some instances, other type of saws such as high‐speed burr, portable circular power saw, and surgical blade have been used for this purpose.^6^, ^7^, ^11^ In this case, a hand saw was used upon the availability of equipment, which is not a very appropriate tool for the surgery. However, it was able to minimize damage to the soft tissues underneath the surgical site.

Contamination is possible in coeliotomy; thus, the environment and instruments should be sterilized prior to the surgery. It is suggested to perform a thorough lavage of the coelomic cavity with lukewarm saline to minimize contamination after gastrotomy or enterotomy.^7^, ^11^ However, in this case lavage was not performed because the first author had taken sufficient precautions to avoid leaking of gastric contents to the coelomic cavity. Nevertheless, an antibiotic (Enrofloxacin) acting against the enteric commensal bacteria and pathogens, were administered intramuscularly for a week post‐surgically, to prevent secondary infections following the surgery.

Turtles are ectothermic animals, therefore, temperature should be monitored and maintained especially after administration of anesthetic medicines. Warm air blankets, which provide homogenous heat without skin burns can be used to administer heat, post‐surgically.^6^ As the surgery was performed in a tropical country with ambient temperature around 27°C in a non‐air‐conditioned room, the turtle was covered with an ordinary surgical drape, which was sufficient to maintain the body temperature.

The clutch size and number of clutches per year show vast variation between chelonian species. Turtle in this case, had five eggs in its abdomen and laid them one at a time. This sub species belongs to the species of *Melanochelys trijuga*, which have two to six individual clutches of eggs per year. The number of clutches depends on factors, such as stress, nutrition status, environmental temperature, humidity, suitable place for nesting, and other reproductive functions.^3^, ^13^ However, this turtle had to face many factors that may have disturbed its natural egg laying. Two days after surgery, the turtle showed signs of normal behavior, such as climbing, perimeter walking, and pre‐nesting. Therefore, it was introduced to a new cage with a sand layer of around six inches height. It started laying few days after the surgery and laid all five eggs within 2 months.

In this case, the Plaster of Paris was used on the incision to avoid contamination of the wound, as a waterproof appropriate sealer was not available. However, later it was understood that a porous non‐watertight material is not suitable for the purpose, thus, it was removed, and epoxy was applied. Epoxy resins and low‐temperature veterinary acrylics (polymethylmethacrylate) have been widely used for chelonion plastron closures and shell repairs.^6^

Transplastron surgeries are less commonly performed in foreign body obstructions in the stomach, compared to axillary approach,^14^ however, as this turtle was a small (weight 2.77 kg) freshwater species, the surgical site of the axillary approach could be too narrow^15^ to perform the surgery, without sophisticated and tiny instruments and an experienced skilled surgeon. Moreover, when the thread can be observed through the oral cavity or cloaca, prognosis can be poor when it is not addressed soon, as the thread inside can cause intestinal plication, which could lead to intestinal damage, blockage, dilatation, and necrosis with potential fatal sequelae.^16^, ^17^

Ingestion of hooks and other dangerous metallic items by chelonians living in contact with humans is a common occurrence. The novel procedure described in this article, demonstrates that it is possible to safely remove such items by cranial transplastron surgery.

## AUTHOR CONTRIBUTIONS

B. G. S. S. G: Handled the case, performed the surgery, and wrote the manuscript, B. V. P. P: Instructed for surgery and post‐operative management (POM), K. K. S: Instructed for surgery and POM also revised the manuscript, K. P. S: POM, V. P. M. K. A: POM, A. S. F: Revised manuscript and supervised the final draft.

## CONFLICT OF INTEREST

All the authors declare no conflict of interest.

## ETHICAL APPROVAL

As the surgery was conducted as routine clinical treatment at the Elephant Transit Home wildlife hospital ethical approval was not necessary.

## CONSENT

The patient is a rescued wild animal from the Uda Walawe National (UW) park in Sri Lanka and was treated at the rehabilitation centre, The Elephant Transit Home (ETH, UW) Sri Lanka. As the veterinarian in charge of the case at the ETH, UW, the primary author gave consent for treatment.

## Data Availability

The data that support the findings of this study are available from the corresponding author upon reasonable request.
